# Supplementary material for: Labeling of nascent RNA in the C. elegans intestine
Source: PLoS One. 2026 Feb 9;21(2):e0341598. doi: 10.1371/journal.pone.0341598 (PMC12885377; doi:10.1371/journal.pone.0341598)
Supplement: S1 Table — (DOCX) [file pone.0341598.s002.docx]

S1 Table for "Labeling of Nascent RNA in *C. elegans* Intestine"

List of *C. elegans* strains used in this study.

| Name | Description | Genotype | Additional Information | Source |
| --- | --- | --- | --- | --- |
| WLW92 | nucl-1::split-gfp | muIs253 [eft-3p::sfGFP1-10::unc-54 3'UTR + Cbr-unc-119(+)] II; nucl-1(sam132[NUCL1::M3]) IV | cross between  [DUP243](https://doi.org/10.1038/s41467-022-34225-5) [1] and [CF4587](https://cgc.umn.edu/strain/CF4587) [2] | This study |
| WLW93 | garr-1::split-gfp | muIs253 [eft-3p::sfGFP1-10::unc-54 3'UTR + Cbr-unc-119(+)] II; garr-1(sam149 [GARR-1::M3])IV | cross between  [DUP250](https://doi.org/10.1038/s41467-022-34225-5) [1] and [CF4587](https://cgc.umn.edu/strain/CF4587) [2] | This study |
| WLW3 | rpoa-2::gfp | ptnsIs053[pCPB155;pCPB157-No1] | Fluorescent tag integrated at endogenous locus using CRISPR-Cas9 | C. P. Brangwynne |
| WLW122 | dao-5::gfp | dao-5(ptnIs050[dao-5::gfp]) I; nucl-1(sam132[NUCL1::M3]) IV | cross between WLW92 and dao-5::gfp CRISPR line [3] | This study |

1. Spaulding EL, Feidler AM, Cook LA, Updike DL. RG/RGG repeats in the C. elegans homologs of Nucleolin and GAR1 contribute to sub-nucleolar phase separation. Nat Commun. 2022;13: 6585. doi:10.1038/s41467-022-34225-5

2. Goudeau J, Sharp CS, Paw J, Savy L, Leonetti MD, York AG, et al. Split-wrmScarlet and split-sfGFP: tools for faster, easier fluorescent labeling of endogenous proteins in Caenorhabditis elegans. Genetics. 2021;217: iyab014. doi:10.1093/genetics/iyab014

3. Berry J, Weber SC, Vaidya N, Haataja M, Brangwynne CP. RNA transcription modulates phase transition-driven nuclear body assembly. Proc Natl Acad Sci USA. 2015;112. doi:10.1073/pnas.1509317112
