# Supplementary material for: Labeling of nascent RNA in the C. elegans intestine
Source: PLoS One. 2026 Feb 9;21(2):e0341598. doi: 10.1371/journal.pone.0341598 (PMC12885377; doi:10.1371/journal.pone.0341598)
Supplement: Attachment — Submitted filename: 251222_Response to Reviewers.docx [file pone.0341598.s005.docx]

We thank the reviewers for their thoughtful and constructive feedback. Based on their helpful suggestions, we have revised our manuscript to include new experimental data, as well as clarifications to the text and figures. Below, we provide a point-by-point response to accompany the revised manuscript.

Reviewers' comments:

Reviewer's Responses to Questions

**Comments to the Author**

1. Does the manuscript report a protocol which is of utility to the research community and adds value to the published literature?

Reviewer #1: Yes

Reviewer #2: Yes

2. Has the protocol been described in sufficient detail?

To answer this question, please click the link to protocols.io in the Materials and Methods section of the manuscript (if a link has been provided) or consult the step-by-step protocol in the Supporting Information files.

The step-by-step protocol should contain sufficient detail for another researcher to be able to reproduce all experiments and analyses.

Reviewer #1: Yes

Reviewer #2: Yes

3. Does the protocol describe a validated method?

The manuscript must demonstrate that the protocol achieves its intended purpose: either by containing appropriate validation data, or referencing at least one original research article in which the protocol was used to generate data.

Reviewer #1: Yes

Reviewer #2: Yes

4. If the manuscript contains new data, have the authors made this data fully available?

The [PLOS Data policy](http://track.editorialmanager.com/CL0/http:%2F%2Fwww.plosone.org%2Fstatic%2Fpolicies.action%23sharing/1/010f019a1a021187-0cdc7a20-7085-4bb9-89ec-87a64ecbe995-000000/uXOkYr9jQZsQrkOa3QNz_ZtMKsRJRbukUiet5d3ml54=234) requires authors to make all data underlying the findings described in their manuscript fully available without restriction, with rare exception (please refer to the Data Availability Statement in the manuscript PDF file). The data should be provided as part of the manuscript or its supporting information, or deposited to a public repository. For example, in addition to summary statistics, the data points behind means, medians and variance measures should be available. If there are restrictions on publicly sharing data—e.g. participant privacy or use of data from a third party—those must be specified.

Reviewer #1: Yes

Reviewer #2: Yes

**5. Is the article presented in an intelligible fashion and written in standard English?**

PLOS ONE does not copyedit accepted manuscripts, so the language in submitted articles must be clear, correct, and unambiguous. Any typographical or grammatical errors should be corrected at revision, so please highlight any specific errors that need correcting in the box below.

Reviewer #1: Yes

Reviewer #2: Yes

6. Review Comments to the Author

Please use the space provided to explain your answers to the questions above. You may also include additional comments for the author, including concerns about dual publication, research ethics, or publication ethics. (Please upload your review as an attachment if it exceeds 20,000 characters)

Reviewer #1: This paper reports a new method for detecting nascent ribosomal RNA (rRNA) in C. elegans intestinal cells. To overcome the impermeability of the C. elegans cuticle, the intestine is removed by dissection for incubation with EU, a uridine derivative that can be labeled by click chemistry for quantitative light microscopy. The method is clearly described and accompanied by a detailed protocol posted at protocols.io. Quantitative imaging detects a robust EU signal in the nucleolus, the established site or rRNA synthesis. RNAi of a canonical transcription factor for rRNA expression depletes the nucleolar EU signal lending strong support to the hypothesis that this method reliably detects nascent rRNA. Lastly, live cell markers (GFP) for nucleolar compartments appear to confirm that rRNA is largely localized to the fibrillar zone (FZ) (see below). This manuscript offers a useful and well-validated protocol for measuring rRNA in the C. elegans intestine and could be potentially useful for detection of nascent RNA in other C. elegans dissected tissues or isolated cells.

Minor Revisions:
1. A key finding is that rRNA transcripts are localized to the nucleolar fibrillar zone (FZ). Figure 3B quantifies co-localization (Spearman correlation coefficient) of the EU-594 signal with FZ markers DOA-1 and GARR-1 vs the granular zone (GZ) marker NUCL-1. Student’s t-test shows no significant difference (ns) between DOA-1 and GARR-1 but no statistics are reported for a comparison of NUCL-1 vs DOA-1 and GARR-1. This statistical test is important for substantiating the authors’ conclusion that the “majority of nascent rRNA transcripts localize to the fibrillar zone.“

We performed two-way ANOVA and Tukey-Kramer test on these data and now include p-values for each pairwise comparison (Fig 3). Consistent with our original conclusion, there is no statistical difference (p > 0.05) between the Spearman correlation coefficients for EU signal with DAO-5 and GARR-1. We now explicitly report statistical differences for all other marker pairs: p < 0.001 for NUCL-1 vs. RPOA-2, DAO-5, and GARR-1, and p < 0.01 for RPOA-2 vs. DAO-5 and GARR-1. These results further validate the distinct localization patterns of our FZ (RPOA-2, DAO-5, GARR-1) and GZ (NUCL-1) markers, and support the conclusion that the majority of EU signal localizes to the FZ.

2. Because intestinal cell nuclei are polyploid, it should be possible to detect other classes of nascent transcripts. This finding would provide a useful demonstration of the broad applicability of the protocol.

The reviewer raises an excellent question. To test whether we could detect other classes of nascent RNA, we performed additional experiments in the presence of flavopiridol, a fast-acting inhibitor of RNA polymerase II. Unexpectedly, we saw a significant reduction in EU signal not only in the nucleoplasm, but also in the nucleolus itself. We suspect that this is due to Pol II’s role in transcribing noncoding RNA from the intergenic spacers between rDNA repeats (PMID: 32669707). We have added these results as a supplementary figure (S2 Fig).

3. Need reference for concentration of ActD sufficient to inhibit RNA Pol II (line 154)

We have added two references to support this statement (line 176). The first (PMID: 21922053) gives concentration guidelines for inhibition of Pol I and Pol II primarily in the context of cultured cells. The second (PMID: 38293118) provides an example of actinomycin D used to inhibit Pol II transcription in *C. elegans*.

4. A figure illustrating click chemistry would be useful to readers unfamiliar with this method.

We thank the reviewer for this suggestion. We have added a panel (H) to Figure 1, illustrating the chemical structure of 5-EU and the click reaction.

Reviewer #2: In this manuscript, Gholamalamdari and Weber present a protocol for nascent labelling of RNA in dissected intestines in C. elegans. Historically, the worm cuticle has prevented facile labelling of RNA with nucleoside analogs. Here, the authors solve this problem by extrusion of the intestine, allowing efficient 5-ethynyl uridine (EU) incorporation. The authors provide good evidence for nascent transcript labelling in the nucleolus, demonstrating reduced incorporation in response to perturbations of transcription, such as treatment with actinomycin D or knockdown of tif-1a (RNA polymerase I transcription factor). Subsequently, the authors further use this technique to explore the location of rRNA transcription within the nucleolus, by co-staining nascent RNA with known markers of the Fibrillar Zone (FZ) and Granular Zone (GZ). Their results demonstrate that the EU signal localizes within the FZ.

Overall, I enjoyed reading this protocol and I think it will be a good addition for the C. elegans community interested in studying gene regulation and transcription.

I only have minor comments that would be helpful to aid others interested in trying this technique.

1) It would be good for the authors to provide some rough guidelines/recommendations on how many animals should be dissected for a typical imaging experiment. It’s not clear how much material loss takes place after the manipulations.

We have added the following recommendation to the guideline section of the protocol:

For each biological or technical replicate we recommend dissecting 20 worms. Due to the material loss during the procedure the recovery rate is between 5 to 10 worms for imaging.

2) Do the authors have some lower magnification images of the labelled intestines? Does the nucleolar/rRNA signal make it difficult to observe mRNA in the nucleus or cytoplasm? This would be interesting to know for anyone interested in studying other RNA species in the cell.

We have added lower-magnification images to Figure S1, and provide line profiles to highlight the differences in EU signal among the nucleolus, nucleoplasm, and cytoplasm.

Since Reviewer #1 asked a similar question, we performed additional experiments in the presence of flavopiridol, a fast-acting inhibitor of RNA polymerase II. Unexpectedly, we saw a significant reduction in EU signal not only in the nucleoplasm, but also in the nucleolus itself. We suspect that this is due to Pol II’s role in transcribing noncoding RNA from the intergenic spacers between rDNA repeats (PMID: 32669707). We have added these results as a supplementary figure (S2 Fig).

7. PLOS authors have the option to publish the peer review history of their article ([what does this mean?](https://track.editorialmanager.com/CL0/https:%2F%2Fjournals.plos.org%2Fplosone%2Fs%2Feditorial-and-peer-review-process%23loc-peer-review-history/1/010f019a1a021187-0cdc7a20-7085-4bb9-89ec-87a64ecbe995-000000/fVVLjGg46h8-qSmmklPJWXyYjmnhTk9eFU4zA6kZxuI=234)). If published, this will include your full peer review and any attached files.

If you choose “no”, your identity will remain anonymous but your review may still be made public.

**Do you want your identity to be public for this peer review?** For information about this choice, including consent withdrawal, please see our [Privacy Policy](https://track.editorialmanager.com/CL0/https:%2F%2Fwww.plos.org%2Fprivacy-policy/1/010f019a1a021187-0cdc7a20-7085-4bb9-89ec-87a64ecbe995-000000/r2CIcaqbNzyG3xjWzKgfVRsb4CgqjOokZy3-8vZzUHY=234).

Reviewer #1: No

Reviewer #2: No
